# Classification of Processing Damage in Sugar Beet (*Beta vulgaris*) Seeds by Multispectral Image Analysis

**DOI:** 10.3390/s19102360

**Published:** 2019-05-22

**Authors:** Zahra Salimi, Birte Boelt

**Affiliations:** Department of Agroecology, Aarhus University, 4200 Slagelse, Denmark; z.salimi@agro.au.dk

**Keywords:** machine vision, mechanical damage, prediction model, seed quality, seed polishing

## Abstract

The pericarp of monogerm sugar beet seed is rubbed off during processing in order to produce uniformly sized seeds ready for pelleting. This process can lead to mechanical damage, which may cause quality deterioration of the processed seeds. Identification of the mechanical damage and classification of the severity of the injury is important and currently time consuming, as visual inspections by trained analysts are used. This study aimed to find alternative seed quality assessment methods by evaluating a machine vision technique for the classification of five damage types in monogerm sugar beet seeds. Multispectral imaging (MSI) was employed using the VideometerLab3 instrument and instrument software. Statistical analysis of MSI-derived data produced a model, which had an average of 82% accuracy in classification of 200 seeds in the five damage classes. The first class contained seeds with the potential to produce good seedlings and the model was designed to put more limitations on seeds to be classified in this group. The classification accuracy of class one to five was 59, 100, 77, 77 and 89%, respectively. Based on the results we conclude that MSI-based classification of mechanical damage in sugar beet seeds is a potential tool for future seed quality assessment.

## 1. Introduction

Monogerm sugar beet seed is a single achene with the outer layer or pericarp composed of lignified cells. During post-harvest processing and conditioning, this corky tissue is rubbed off by passing the seeds through specialized machines where size and shape become uniform [1]. However, the processed seeds are still heterogeneous in quality and need further improvement in order to meet market requirements. In sugar beet, there is a high demand for a uniform and high field emergence. Biological and mechanical factors like empty or partially filled seeds, broken embryos or fractures of the pericarp can affect seed performance in the field [2]. 

Sugar beet is a biennial plant, and seeds are produced in the second growing season after a primary inflorescence induction by low temperature and short days during the autumn and winter followed by a secondary induction by longer days in spring [3]. During the spring, the plants undergo bolting and the reproductive organs develop. Sugar beet has an indeterminate flowering pattern, which results in seeds varying in maturity and size at harvest time. The seed size generally declines gradually moving up the spike.

Sugar beet seeds are best produced in areas with temperatures ranging from 15 to 20 °C during flowering and ripening, and with infrequent rainfall, in particular during ripening. The optimal locations in Europe are southern France and northern Italy.

After harvest, seeds are processed in order to improve the physical purity of the seed lot, and the seeds are subsequently polished. The main purpose of polishing is to make seeds uniform for pelleting, to improve water uptake and to enhance germination by the removal of growth inhibitors, which are located in the pericarp [4,5]. Seed appendages are rubbed off in the cylindrical frame of the polisher by the abrading action of revolving brushes. As with any mechanical operation, excessive processing can cause damage to the seed coat [6,7]. Damage can be extended to the interior parts of the seed and affect physiological processes. Regardless of damage intensity, mechanical injuries decrease the seed longevity, expose the seed to the infection, reduce yield and reduce germination [7]. 

Mechanically damaged seeds should not be used in the pelleting unless the injury is very small. Due to their sensitivity to water uptake [4], damaged seeds may result in heterogeneous field performance, and there is also evidence from soybean, sweet corn and maize that the damaged seeds are more likely to produce abnormal seedlings [7,8,9,10]. Therefore, it is important to identify mechanically damaged seeds in order to discard them, but also in order to improve our knowledge about the specific procedure causing the injuries. This knowledge may help in adjusting the procedure and prevent future excessive mechanical disturbance [4,11].

Determination of damaged sugar beet seeds is currently based on the visual inspection of different physical quality attributes. This is a subjective method relying on trained personnel; it is time-consuming and therefore costly. Non-destructive, reliable and preferably rapid evaluation techniques are required to ensure the quality of the seeds at the different stages of processing, storage, transport and seeding [12,13,14]. Recent advantages have been obtained by machine vision, which provides an automated, rapid and non-destructive identification [15].

Machine vision is the use of modern camera technology and image analysis. Fast progression in this field has led to camera systems today that are capable of rendering high-resolution multispectral imaging (MSI) in which each pixel contains the information of reflected or absorbed light of various wavelengths from the surface of the object under investigation. This new technology can emulate human vision and provide documentation for further evaluation [11,12,13]. 

MSI is a combination of imaging and spectroscopy, which offers the advantage over traditional imaging methods by the simultaneous gathering of spatial and spectral information of the object surface [16,17,18]. MSI is predicted to become a useful non-destructive tool in seed testing [19,20,21], with increased automation and minimum use of specialized inspectors [22]. As the mechanical damage appears on the seed surface, the analyses can be done in a simple and rapid way without seed pre-treatment [16,17,18]. The ability of this technique to include multiple variables provides a great potential for seed quality testing, and this approach has been applied in various seed related studies [18].

It is important for the seed industry to be able to classify various damage types in seed lots with mixed mechanically damaged seeds, as each damage type affects seed performance differently. Therefore, the objective of this study was to establish a model for classifying different damage types of polished sugar beet seeds in a mixed sample by means of MSI.

## 2. Materials and Methods

Two groups of sugar beet (*Beta vulgaris*) seeds with varying degrees of mechanical damage were provided by MariboHilleshög, Holeby, Denmark. One group, referred to as the calibration set, consisted of five samples, each with a specific damage class. There were 301 seeds in total in these five samples. Another group, referred to as the test set, consisted of 18 samples, where the seeds of each sample had mixed damage types. Each sample was from an individual variety, and overall 200 seeds were in this group. Samples from the calibration set were used to train the classification model, and samples from the 18 varieties with mixed damage were used to validate the model.

**Figure 1:**
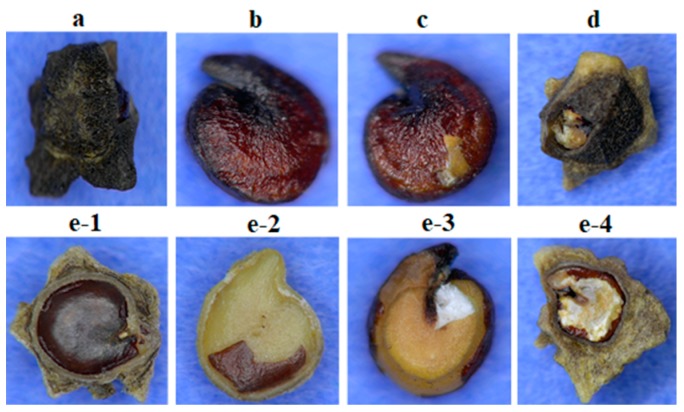
Five damage classes (**a**–**e**) of sugar beet seeds: (**a**). Partially broken pericarp and/or outer testa (class 1), (**b**). Completely broken pericarp and outer testa (class 2), (**c**). Fractured pericarp and outer testa, partially crushed inner testa with sound embryo (class 3), (**d**). partially broken pericarp and/or outer testa, damaged inner testa with intact embryo (class 4), (**e1–4**). Different types of severe damages to the embryo or seeds without any embryo like the pericarp or outer testa (class 5).

The damage classes are defined as: 

(1) Seeds with a partially broken pericarp and/or outer testa and without any damage to the inner testa. These seeds are used for pelleting, and hence specific consideration should be carried out in classifying seeds in this class as they will be part of the commercial seed lot (Figure 1a). 

(2) Intact inner testa covering the seed, pericarp and outer testa are completely broken. These seeds are either discarded or in some cases used for pelleting with the risk of producing abnormal seedlings (Figure 1b). 

(3) Both pericarp and outer testa are fractured and the inner testa is partially crushed, but the embryo is sound. Seeds with this damage type may produce abnormal seedlings and are usually discarded (Figure 1c). 

(4) Seeds with partially broken pericarp and/or outer testa and with damage to the inner testa; embryo intact. Seeds of this type may also produce abnormal seedlings and are usually discarded (Figure 1d). 

(5) Seeds without embryo or with severe damage to the embryo (Figure 1e). Seeds of this damage type will not produce a normal seedling and are discarded. 

Multispectral images of all seed samples were taken by a VideometerLab 3 instrument (Videometer A/S, DK-2970, Hørsholm, Denmark). The instrument consists of a sphere containing 19 light emitting diodes in the wavelengths 375, 405, 435, 450, 470, 505, 525, 570, 590, 630, 645, 660, 700, 780, 850, 870, 890, 940 and 970 nm. All images were acquired in one sequence and had a resolution of 2056 by 2056 pixels. 

Initially images were taken of both sides of the seeds (ventral and dorsal side) from the calibration sample set to derive the characters of each damage class by use of VideometerLab software. Image analysis algorithms are readily available in this software and the software guidelines includes recommendations for the specific type of study.

**Figure 2:**
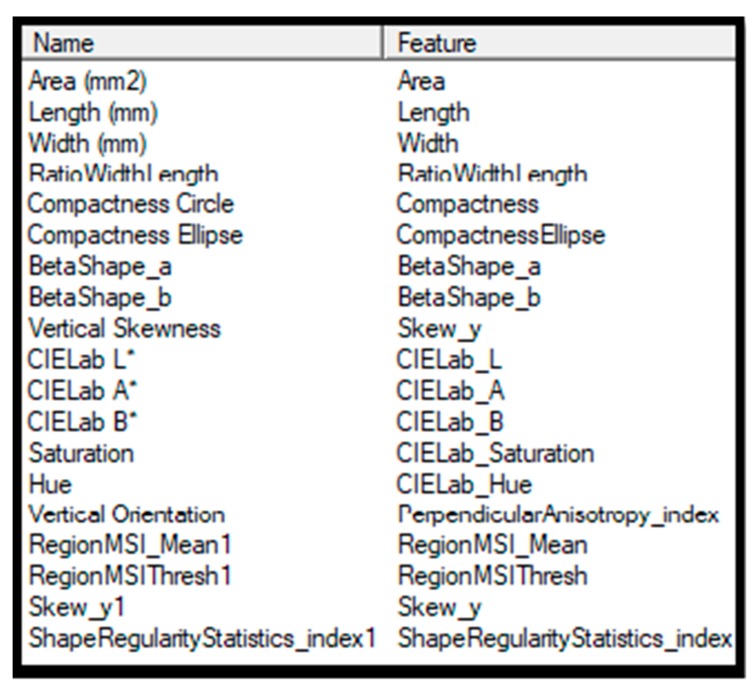
List of the 19 extracted variables from multi spectral images.

A normalized Canonical Discriminant Analysis (nCDA) was applied to calculate the transformation model of the five damage classes. nCDA helps to choose the optimal band normalization on the pixel level to be able to map the data to a space where the ratio between variance between groups and variance within groups is maximized. Following the image analysis, 19 variables related to the seed shape, color and binary features were extracted in an Excel file from the calibration set (Figure 2). These variables were chosen through the VideometerLab company recommendation. The statistical analysis was carried out using the VideometerLab software version 3.6.9 (Videometer A/S) and SAS (version 9.3).

Subsequent to extracting each seed′s information from multispectral images, a combination of PROC FACTOR and PROC SCORE command was used in the SAS 9.3 environment to develop a supervised classification model. The SCORE procedure used the extracted coefficients from the calibration data set as the scoring tool for the validation data set. The output data set consisted of linear combinations between the coefficients and the validation data set. The FACTOR procedure was applied for extracting coefficients from the calibration data set, since scoring coefficients are normalized in order to give united variance principle component scores [23]. Thereafter, a classification model was designed by the use of derived scores from the calibration data set with 302 seeds (60% of sample), and it was applied on the validation data set of samples with 200 seeds of unknown mixed damage type (equal to 40% of total). Derived scores from the calibration data set and both sides of the seeds in the validation sample, mean and standard deviation of classes were evaluated for identifying the range of each damage class. Mean plus or minus the standard deviation defines the minimum and maximum score range of each class. To correct the overlaps or the gaps between the classes, if existing, the maximum of the previous class was chosen to be the minimum of the next class. Ventral and dorsal side scores were compared to the ranges and classified. 

In order to check the accuracy of classification, seeds were investigated visually by assessing each seed with a TAGARNO MAGNUS HD TREND (Horsens, Denmark) digital microscope. Finally, the identified class of each seed was compared with the model prediction output. The classification accuracy, false negative and false positive percentage were calculated with Equations (1)–(3), respectively:

(1) $$Classification\;accuracy\left(\%\right)=\frac{Number\;of\;seeds\;correctly\;classified\;by\;the\;model}{Total\;number\;of\;seeds}\times 100$$

(2) $$False\;negative\left(\%\right)=\frac{False\;negatives}{Total\;number\;of\;evaluated\;seeds}\times 100$$

(3) $$False\;positive\left(\%\right)=\frac{False\;positives}{Total\;number\;of\;evaluated\;seeds}\times 100$$

False positive is defined as the number of seeds that the model classified in a specific class but should not be there. False negative is the number of seeds that belong to a class but the model did not classify them to this class.

## 3. Results and Discussion

Mechanical damage during processing may affect the seed quality and germination percentage, and seedling vigor is found to correlate with this damage [24]. In this study, five different types of damage to sugar beet seeds were evaluated (Figure 1). The seed coat may be damaged without affecting the actual seed and/or the embryo (Figure 1a), or the polishing operation may severely crush the embryo, leaving no chance of a normal seedling to develop (Figure 1(e1–e5)).

Mechanical damage of the seed surface has been investigated with techniques such as X-ray, visible/near-infrared hyperspectral imaging, spectroscopy and thermal imaging [14,25,26,27,28]. In recent years, MSI has been applied in classification models to discriminate between cultivars and fungal infection, and in viability prediction models [16,17,20,29,30]. 

**Figure 3:**
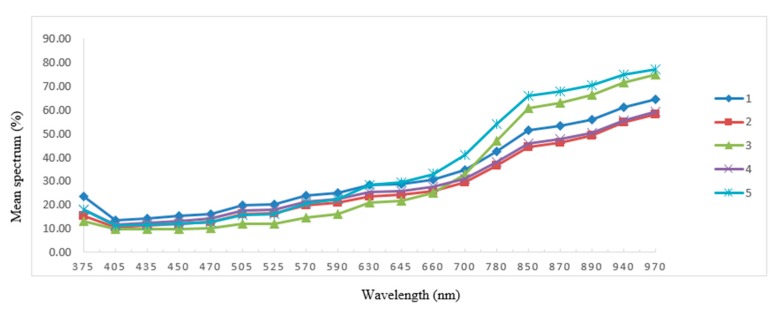
Mean spectrum (percentage) of 19 wavelengths (nm) in five damage classes (1–5) of sugar beet.

**Figure 4:**
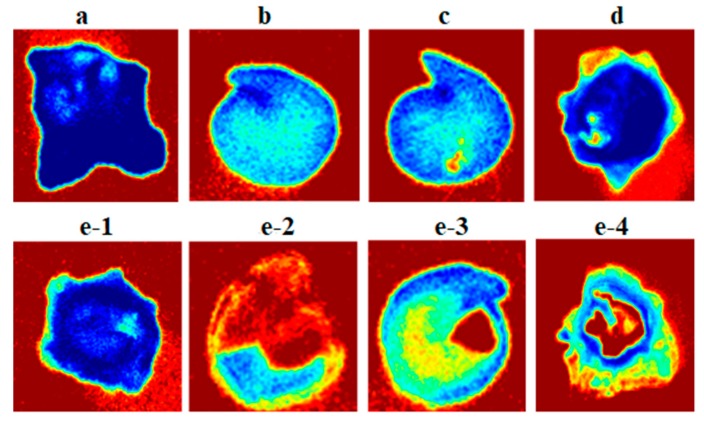
The nCDA-transformed images of mechanical damage class (1–5) in sugar beet seeds.

The mean spectrum of the five damage classes was derived from the 19 wavelengths employed in the VideometerLab instrument (Figure 3). In general, the mean spectrum showed very little diversity in the visible wavelength region (below 709 nm), whereas more diversity was found in the Near InfraRed (NIR) wavelengths. Classification of damage types by use of the raw images of sugar beet seeds is not a readily available task; however, the designed model developed from the 19 variables presented a way to classify the processing damage of sugar beet seeds. The eventuated transformation of RGB images from Figure 1 is illustrated in Figure 4. Although classes are similar in color, texture and shape, the model classified damage by emphasizing the severity of the damage to the inner testa and embryo. 

Figure 4 shows the nCDA-transformed images of the five damage classes. The blue range of color in the transformed images indicates the dark part of the seed, and the red range is the indicator of the lighter part. Areas with high intensity of red color show the probably damaged parts of the embryo or interior segments of the outer testa. Therefore, the position and amount of red color is the indicator of damage class.

The statistical analysis of data from the MSI transformation model, by use of the standardized scoring coefficients from the FACTOR analysis, presented a high influence of four color variables derived from the classification model. The output of applying this model to 200 seeds from 18 different varieties was 163 correct and 37 misclassified seeds, which is equal to 82% accuracy.

In a recent study, Sendin et al. [30] classified defects in white maize kernels by MSI and reached 83 to 100% accuracy, depending on the type of damage, but this study did not include mechanical damage during processing. Instead, it included damage from rodents where the obtained classification accuracy was 89%. Damage types were not illustrated, but it assumingly affected the seed morphology and shape. In our study, it is observed from Figure 1 that the difference between damage classes 2 and 3 (Figure 1b,c) may be very small fractures of the seed surface.

**Figure 5:**
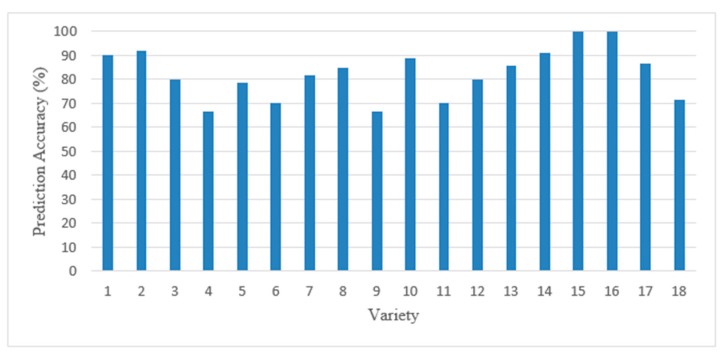
The prediction accuracy (percentage) of each of the 18 varieties in response to the classification model.

Among the 18 varieties, the prediction accuracy ranged from 67 to 100% (Figure 5). There were 10 varieties with more than 80% correct classification, and two varieties that had a prediction accuracy lower than 70%. This variability in prediction accuracy may be caused by differences in seed structure among the varieties, leading to different susceptibility to processing damages, and some varieties typically had only small damages that were harder to identify. Further, differences in pericarp color among varieties also affected prediction accuracy.

For each of the five damage classes, the classification accuracy was 59%, 100%, 77%, 77% and 89% for classes 1–5, respectively (Table 1). Seeds of class 1 are the only ones that can be used for seed enhancement treatments. As it is critical to choose the correct seeds for this class, the model was built to omit seeds rather than to allow false positive seeds in this group, and moreover, components such as pericarp or outer testa could be pelleted and considered as a real seed. Therefore, avoidance of placing these components in class 1 is necessary. 

Due to the high similarity of damage types, identifying them is quite hard, especially between classes with color or size similarities. Since class 5 consists of severely damaged seeds, this class separation seems more rigid for the model. Moreover, a high total number of damaged seeds in class 4 explains the high false negative percentage in this class (Table 1). 

Overall, this study displayed the potential of MSI in classification of various damage types, without additional analytical evaluation. Further studies using a wider range of samples may be required, even though this scale was appropriate enough to show the strength of the method. This study demonstrates MSI as a tool for the identification of mechanical damage from polishing during processing, and hence demonstrates MSI as a tool in seed quality assessment.

## 4. Conclusions

A classification model based on MSI derived information about surface characteristics and multivariate data analysis enabled discrimination into five damage classes with 82% overall accuracy. Currently these damages are identified by visual inspections, which are time consuming and require trained seed analysts. However, a professional analyst may still have errors in the classification of these types of damages, and the reproducibility between different analysts may not be sufficiently high. Multispectral imaging as a fast means of classification will give the chance to repeat the sample categorization and obtain reproducible results.

The future perspective of generating a prediction model for seed damage classification should focus on improving the model function accuracy by doing a stepwise analysis and including more and diverse seed samples. 

Indeed, further studies may introduce innovative ways of developing MSI classification models for seed quality assessment.

## Figures and Tables

**Table 1:** Number of misclassified seed in each damage group, classification accuracy, false negative and false positive percentage for each damage class (1–5).

Class	Misclassified					Classification Accuracy (%)	False Negatives (%)	False Positives (%)
	1	2	3	4	5			
**1**	-	0	1	4	2	59	3	1.5
**2**	0	-	0	0	0	100	0	1.5
**3**	1	2	-	0	0	77	1.5	2
**4**	2	1	1	-	13	77	8.5	8.5
**5**	0	0	2	8	-	89	5	5.5
